# Arsenite Stress Down-regulates Phosphorylation and 14-3-3 Binding of Leucine-rich Repeat Kinase 2 (LRRK2), Promoting Self-association and Cellular Redistribution*

**DOI:** 10.1074/jbc.M113.528463

**Published:** 2014-06-18

**Authors:** Adamantios Mamais, Ruth Chia, Alexandra Beilina, David N. Hauser, Christine Hall, Patrick A. Lewis, Mark R. Cookson, Rina Bandopadhyay

**Affiliations:** From the ‡ Reta Lila Weston Institute of Neurological Studies, University College London Institute of Neurology, London WC1N 1PJ, United Kingdom,; the § Department of Molecular Neuroscience, University College London Institute of Neurology, London WC1N 3BJ, United Kingdom,; the ¶ Cell Biology and Gene Expression Section, Laboratory of Neurogenetics, NIA, National Institutes of Health, Bethesda, Maryland 20892,; the ‖ Department of Neuroscience, Georgetown University Medical Center, Washington, D. C. 20057,; the ** Brown University/National Institutes of Health Graduate Partnership Program, Department of Neuroscience, Brown University, Providence, Rhode Island 02912, and; the ‡‡ School of Pharmacy, University of Reading, Whiteknights, Reading RG6 6AP, United Kingdom

**Keywords:** Leucine-rich Repeat Kinase 2 (LRRK2), Neurodegenerative Disease, Oxidative Stress, Parkinson Disease, Phosphorylation, Arsenite

## Abstract

Mutations in the gene encoding leucine-rich repeat kinase 2 (LRRK2) are a common genetic cause of Parkinson disease, but the mechanisms whereby LRRK2 is regulated are unknown. Phosphorylation of LRRK2 at Ser^910^/Ser^935^ mediates interaction with 14-3-3. Pharmacological inhibition of its kinase activity abolishes Ser^910^/Ser^935^ phosphorylation and 14-3-3 binding, and this effect is also mimicked by pathogenic mutations. However, physiological situations where dephosphorylation occurs have not been defined. Here, we show that arsenite or H_2_O_2_-induced stresses promote loss of Ser^910^/Ser^935^ phosphorylation, which is reversed by phosphatase inhibition. Arsenite-induced dephosphorylation is accompanied by loss of 14-3-3 binding and is observed in wild type, G2019S, and kinase-dead D2017A LRRK2. Arsenite stress stimulates LRRK2 self-association and association with protein phosphatase 1α, decreases kinase activity and GTP binding *in vitro*, and induces translocation of LRRK2 to centrosomes. Our data indicate that signaling events induced by arsenite and oxidative stress may regulate LRRK2 function.

## Teaser

**Background:** LRRK2 mutations are causative for Parkinson disease, but regulation of LRRK2 remains elusive.

**Results:** Arsenite induces loss of LRRK2 Ser^910^/Ser^935^ phosphorylation and 14-3-3 binding, increased self-association, attenuated kinase activity and GTP binding, and translocation to centrosomes.

**Conclusion:** LRRK2 is regulated by arsenite-induced signaling and oxidative stress.

**Significance:** Understanding LRRK2 regulation will provide novel approaches toward developing therapeutic tools targeting LRRK2 activity.

## Introduction

Parkinson disease (PD)^4^ is a neurodegenerative disorder characterized by rigidity, tremor, postural instability, and other symptoms, and for the familial form (around 10% of cases), a number of causative genetic factors have been identified (1–3). Mutations in the gene coding for LRRK2 are a frequent cause of autosomal dominant familial PD (4, 5), present in about 5% of familial cases and 1–2% of apparently sporadic cases (6). LRRK2 is a large protein with two enzymatic domains: a GTPase domain (ROC; Ras of complex proteins) that is thought to operate in tandem with the adjacent COR (C-terminal of ROC) domain and a serine/threonine protein kinase domain (7, 8). The protein also contains N-terminal ankyrin-like repeats, leucine-rich repeats, and a WD40 domain (9). The most prevalent mutation, G2019S, enhances the kinase activity of LRRK2 and induces neuronal cell death *in vitro* (10, 11) and *in vivo* (12).

LRRK2 is constitutively phosphorylated at Ser^910^ and Ser^935^, and phosphorylation is required for binding of 14-3-3 proteins, which in turn control the cellular localization of LRRK2 (13). Several pathogenic mutants, including R1441C and Y1699C, show markedly diminished phosphorylation at these sites and decreased 14-3-3 interaction (14). Additionally, pharmacological inhibition of LRRK2 kinase activity leads to decreased Ser^910^/Ser^935^ phosphorylation, resulting in a concomitant decrease in 14-3-3 binding and inducing translocation of LRRK2 into discrete cytoplasmic pools (15). However, kinase-dead variants of LRRK2 do not show loss of Ser^910^/Ser^935^ phosphorylation (15) or altered cellular localization (16) under IN-1 treatment. This suggests a feedback loop whereby inhibition of active LRRK2 kinase alters activity of unidentified kinases or phosphatases, which impacts the phosphorylation status of LRRK2 itself. A recent study has suggested that Ser^910^/Ser^935^ phosphorylation is mediated by IκB kinases in response to activation of the Toll-like receptor pathway, but this is not blocked by inhibition of LRRK2 kinase activity (17). These studies suggest the existence of diverse pathways that regulate LRRK2 phosphorylation status not related to its kinase activity, which remain largely unexplored (18).

Oxidative stress is believed to play an important role in the pathogenesis of PD, with the parkinsonism-linked genes *PARK2*, *PINK1*, and *DJ1* being associated with mitochondrial dysfunction and increased reactive oxygen species-linked cellular effects (reviewed in Ref. 19). Dopaminergic cell loss, characteristic of PD, can be mimicked *in vivo* by exposure to toxins such as 1-methyl-4-phenyl-1,2,3,6-tetrahydropyridine (MPTP) (20, 21) or rotenone, which inhibit Complex I of the mitochondrial respiratory chain (22, 23). LRRK2 has been linked to protection from mitochondrial stress through interaction with kinases of the mitogen-activated protein kinase family (24), whereas recent studies suggest that G2019S LRRK2 causes uncoupling of mitochondrial oxidative phosphorylation (25, 26).

Based on these data, we hypothesized that LRRK2 is involved in the oxidative stress response and explored this using the oxidative stressor arsenite. We assessed changes in phosphorylation, self-association, kinase activity, GTP binding, and cellular localization of LRRK2 under arsenite stress. We found that both arsenite and H_2_O_2_-induced stress promoted the loss of LRRK2 phosphorylation at sites Ser^910^/Ser^935^ in stable inducible expression cell lines as well as of endogenous LRRK2 in a lymphoblastoid cell line, whereas this was rescued by inhibition of protein phosphatases. WT LRRK2 and the variants R1441C, D2017A (kinase-dead), and G2019S responded to oxidative stress in a similar manner with loss of constitutive phosphorylation and loss of 14-3-3 binding. Arsenite stress induced LRRK2 self-association as well as accumulation of very high molecular mass forms of LRRK2. Treatment with arsenite resulted in attenuation of kinase activity in LRRKtide phosphorylation and autophosphorylation assays and a decrease in LRRK2 binding to GTP *in vitro*. Arsenite stress also induced LRRK2 translocation to ubiquitin proteasomal centers localizing at centrosomes. Our data collectively support a role of oxidative stress in modulating LRRK2 activity and suggest a sequence of signaling events induced by arsenite stress.

## EXPERIMENTAL PROCEDURES

### 

#### 

##### Cell Culture, Treatments, and Constructs

Inducible HEK-293T cell lines expressing different GFP LRRK2 variants were grown and cultured as described previously (14). Human lymphoblastoid cells were obtained from the Coriell Institute cell repositories. The 3×FLAG-tagged construct of LRRK2 in pCHMWS plasmid was a gift from Dr. J. M. Taymans (KU Leuven, Belgium) (27). Treatments with oxidative stressors were carried out with the described concentrations of sodium arsenite or H_2_O_2_ for 45 min. The LRRK2 inhibitor LRRK2-IN1 was used at 1 μm for 2 h. MG132, lactacystin, and nocodazole were used at 50 μm for 6 h. In the combination treatment experiments, cells were primed with IN-1 for 75 min or with MG132 or nocodazole for 5 h before the addition of arsenite for the last 45 min of the treatment. In the calyculin A experiments, cells were pretreated with 10 nm calyculin A for 15 min before the addition of arsenite, IN-1, or H_2_O_2_ and incubation for an additional 30 min.

##### Co-immunoprecipitation

HEK-293T cells stably expressing GFP WT LRRK2 were transfected with 3×FLAG-LRRK2 variants using Lipofectamine 2000 (Invitrogen) as per the manufacturer's instructions. After 24 h, cells were lysed in buffer containing 20 mm Tris/HCl (pH 7.4), 137 mm NaCl, 3 mm KCl, 10% (v/v) glycerol, 1 mm EDTA, and 0.3% Triton X-100 supplemented with protease inhibitors (Roche Applied Science) and phosphatase inhibitors (Pierce). Lysates were centrifuged at 21,000 × *g*, 4 °C for 10 min, and the supernatants were analyzed for protein concentration (Pierce). 20 μg of total protein from each supernatant was analyzed by SDS-PAGE for expression of the proteins in question. 400 μg of each sample was precleared with protein G beads (Sigma-Aldrich) for 1 h at 4 °C, and subsequently, GFP-LRRK2 was immunoprecipitated with Chromotek-GFP-Trap-agarose resin (Allele Biotech) for 2 h at 4 °C. The GFP-agarose was gently washed six times with buffer containing 20 mm Tris/HCl (pH 7.4), 137 mm NaCl, 3 mm KCl (all from KD Medical), and 0.1% Triton X-100. The washed beads were boiled for 10 min in 4× NuPAGE loading buffer (Invitrogen) supplemented with 1.4 m β-mercaptoethanol and analyzed by SDS-PAGE. Each co-immunoprecipitation was repeated in three independent experiments, and quantification was performed by estimating the ratio of immunoprecipitated binding partner to the amount of LRRK2 construct pulled down on the beads.

##### Size Exclusion Chromatography

Following treatment with sodium arsenite or vehicle control, LRRK2IN-1, or sodium arsenite plus LRRK2IN-1, HEK-293T cells were harvested in PBS supplemented with protease inhibitors and phosphatase inhibitors (Roche Applied Science and Pierce) and lysed using five freeze-thaw cycles in liquid nitrogen. Lysates were centrifuged at 21,000 × *g*, 4 °C for 10 min, and the supernatants were then passed through 0.45-μm filters (Nanosep MF, Pall Life Sciences) by centrifugation at 14,000 × *g* for 3 min to remove any insoluble material. Size exclusion chromatography was performed using a BioAssist G4SW_XL_ column (7.8 mm × 30.0 cm; Tosoh Bioscience) with PBS as the mobile phase as described before (16). The collected fractions were analyzed by SDS-PAGE followed by Western blot analysis for GFP-LRRK2. The distribution of LRRK2 in each fraction was estimated by quantitation densitometry of the bands corrected to the total amount of immunoreactivity in all fractions.

##### Western Blot Antibodies

Standard Western blot protocols were used with the following antibodies: anti-phospho-Ser^910^ LRRK2 (UDD1 15(3)), anti-phospho-Ser^935^ LRRK2 (UDD2 10(12)), anti-phospho-Thr^1410^ LRRK2 (MJFR4-25-5), and anti-LRRK2 (C41-2) from Abcam; anti-phospho-Ser^51^ eIF2α from Epitomics; anti-eIF2α and anti-ubiquitin from Santa Cruz Biotechnology, Inc.; and anti-β-actin from Sigma-Aldrich.

##### GTP Binding Assay

The GTP binding properties of LRRK2 were assessed as described previously (28). Briefly, HEK-293T cells were treated with 0.5 mm sodium arsenite for 45 min and lysed in buffer containing 20 mm Tris/HCl (pH 7.4), 137 mm NaCl, 3 mm KCl, 10% (v/v) glycerol, 1 mm EDTA, and 1% Triton X-100 supplemented with protease inhibitors (Roche Applied Science) and phosphatase inhibitors (Pierce), by 1 h of rotation at 4 °C. Lysates were centrifuged at 21,000 × *g*, 4 °C for 10 min, and supernatants were precleared with agarose beads for 1 h, rotating at 4 °C, followed by centrifugation at 3,000 × *g*, 4 °C for 5 min to separate the lysate from beads. Subsequently, equal amounts of protein were incubated with 30 μl of GTP-agarose (Sigma-Aldrich, G9768) with rotation for 2 h at 4 °C with some samples supplemented with 10 mm GTP as a competitive inhibitor of binding to the beads. GTP-agarose beads were washed three times in lysis buffer, eluted with 4× NuPAGE loading buffer, and analyzed by SDS-PAGE. In a separate experiment, a titration of increasing concentrations of GTP was used to elute LRRK2 from GTP-agarose beads before analysis by Western blot.

##### Kinase Assay

*In vitro* kinase assays were performed as described previously (10, 29). For the LRRKtide assay, FLAG-tagged LRRK2 was transiently expressed in HEK293 FT cells for 24 h, and proteins were purified in lysis buffer (20 mm Tris/HCl (pH 7.4), 300 mm NaCl, 3 mm KCl, 10% (v/v) glycerol, 1 mm EDTA, and 1% Triton X-100 supplemented with protease inhibitors (Roche Applied Science) and phosphatase inhibitors (Pierce) with FLAG M2-agarose beads (Sigma). *In vitro* autophosphorylation kinase assays in the presence of different concentrations of sodium arsenite were performed with 10 nm recombinant GST-LRRK2(970–2527) (Life Technologies) or recombinant GST-CK1α (Signal Chem) in 1× kinase buffer (Cell Signaling), 6 μCi of [^33^P]ATP (3000 Ci/mmol; PerkinElmer Life Sciences), and 10 μm ATP for 30 min at 30 °C. Reactions were terminated by adding 4× NuPAGE loading buffer and analyzed by SDS-PAGE, whereas incorporated ^33^P was detected by autoradiography. In the LRRKtide phosphorylation assay, incorporation of ^33^P was detected by liquid scintillation counting.

##### Immunostaining

HEK-293T cells were seeded at 7 × 10^4^ cells/well on 12-mm coverslips precoated with laminin/poly-d-lysine (Millicell EZ slide, Millipore) and cultured as described before (14). Cells were fixed in 4% (w/v) paraformaldehyde/PBS, blocked in 5% (v/v) FBS in PBS, and stained in blocking solution for 2 h. Primary antibodies were anti-LRRK2 (Abcam), anti-γ-tubulin (Abcam), anti-α-tubulin (Sigma-Aldrich). After three washes in PBS, the cells were incubated for 1 h with FITC- and TRITC-conjugated secondary antibodies and TO-PRO-3 nuclear stain (Invitrogen). After additional washing steps, the cells were analyzed by confocal microscopy (Zeiss LSM 710).

##### Statistical Analysis

Experiments examining phosphorylation levels of LRRK2 under oxidative stress, LRRK2 self-association experiments, and centrosomal localization experiments were analyzed by one-way ANOVA with Tukey's post hoc test. A two-tailed Student's *t* test was used in the GTP binding experiments. All statistical analyses were performed using GraphPad Prism version 4 for Windows (GraphPad Software, San Diego, CA). Mean values ± S.E. are indicated.

## RESULTS

### 

#### 

##### Oxidative Stress Induces Loss of LRRK2 Constitutive Phosphorylation and Impaired 14-3-3 Binding

**FIGURE 1.**
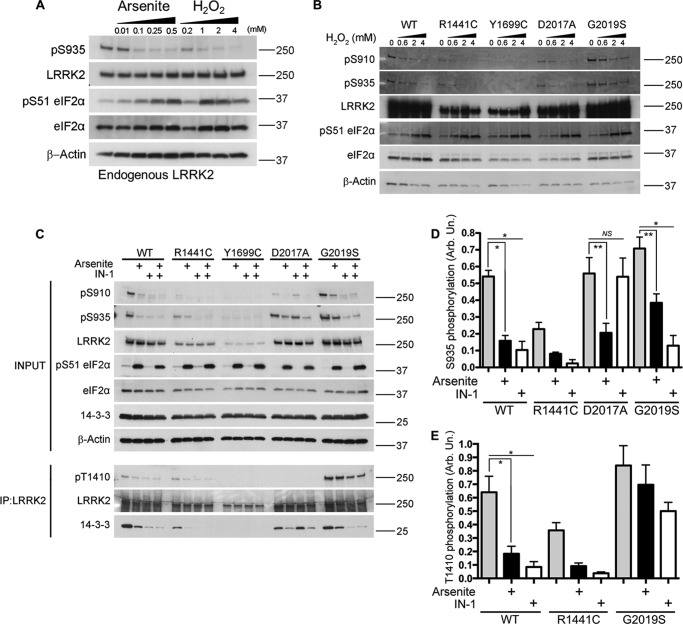
**Arsenite-induced cellular stress promotes dephosphorylation of LRRK2 and loss of 14-3-3 interaction.** *A*, endogenous LRRK2 has decreased Ser^935^ phosphorylation in response to treatment with increasing concentrations of arsenite or H_2_O_2_ in human lymphoblastoid cells. eIF2α phosphorylation at Ser^51^ is induced under the same conditions of cellular stress. *B*, treatment with increasing concentrations of H_2_O_2_ induces loss of Ser^910^ and Ser^935^ phosphorylation of GFP LRRK2 WT and the variants R1441C, D2017A, and G2019S in stable expression HEK-293T cells. *C*, treatment with arsenite (0.5 mm, 45 min) induces LRRK2 dephosphorylation at Ser^910^ and Ser^935^ along with loss of 14-3-3 binding for all variants tested. LRRK2IN-1 induces loss of phosphorylation and 14-3-3 binding in WT, R1441C, and G2019S but not in the kinase-dead D2017A. Phosphorylation at Thr^1410^ is increased in G2019S compared with WT and the other variants. Arsenite treatment induces a significant loss in Thr^1410^ phosphorylation of WT LRRK2. *D*, quantification of loss of Ser^935^ LRRK2 phosphorylation after arsenite treatment; for all LRRK2 constructs where phosphorylation was detectable, this was reduced in the presence of arsenite (one-way ANOVA; Tukey's post hoc test; *, *p* < 0.01; **, *p* < 0.05; *n* = 3 independent experiments/condition). *E*, quantification of loss of Thr^1410^ phosphorylation after arsenite treatment (one-way ANOVA; Tukey's post hoc test; *, *p* < 0.01; *n* = 3 independent experiments/condition). *IP*, immunoprecipitation. *Error bars*, S.E.

To investigate how oxidative stress alters phosphorylation of endogenous LRRK2, we treated human lymphoblastoid cells with arsenite or H_2_O_2_. We observed a concentration-dependent reduction in Ser^935^ phosphorylation with arsenite (>100 μm), and with H_2_O_2_ (>200 μm) (Fig. 1*A*). Arsenite stress promotes translational arrest by mediating phosphorylation of the mammalian translation initiation factor eIF2α. Activation of this cellular stress response was therefore verified by monitoring the concentration-dependent increase in eIF2α phosphorylation under the same conditions (Fig. 1*A*).

To examine whether these effects were also seen with mutant forms of LRRK2, we repeated these experiments in stably expressing LRRK2-inducible HEK-293T cell lines (10, 30, 31). H_2_O_2_ (Fig. 1*B*) or arsenite (Fig. 1*C*) induced loss of Ser^910^/Ser^935^ phosphorylation of WT as well as R1441C, D2017A, and G2019S mutants in a concentration-dependent manner. LRRK2 Y1699C displayed greatly reduced constitutive Ser^910^/Ser^935^ phosphorylation, in agreement with previous studies (14).

To further evaluate the requirement of kinase activity, LRRK2-expressing cells were treated with arsenite in the presence or absence of the LRRK2 kinase inhibitor LRRK2IN-1. As with endogenous LRRK2, treatment with 0.5 mm arsenite caused a reduction in Ser^910^/Ser^935^ phosphorylation for WT, R1441C, G2019S, and the kinase-dead D2017A. Treatment with LRRK2IN-1 induced a comparable loss of phosphorylation but, crucially, not in the kinase-dead D2017A mutant (Fig. 1, *C* and *D*).

We also looked at Thr^1410^ autophosphorylation of immunoaffinity-purified GFP-LRRK2 (Fig. 1, *C* and *E*) (32). We observed increased Thr(P)^1410^ signal in G2019S compared with WT, whereas arsenite treatment induced loss of Thr^1410^ phosphorylation in WT LRRK2 in a similar fashion to treatment with LRRK2IN-1 but had a lesser effect on G2019S Thr(P)^1410^ (Fig. 1*E*).

Loss of Ser^910^/Ser^935^ phosphorylation should decrease LRRK2 binding to 14-3-3 proteins. To test this, we immunoprecipitated LRRK2 from cells expressing WT LRRK2 or mutants after treatment with arsenite and/or LRRK2IN-1. In all cases, loss of Ser^910^/Ser^935^ phosphorylation was accompanied by decreased co-immunoprecipitation of 14-3-3 (Fig. 1*C*).

**FIGURE 2.**
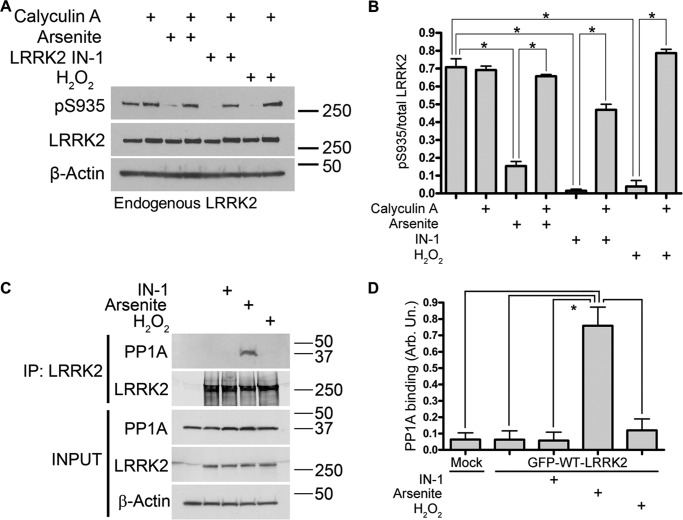
**Calyculin A treatment prevents endogenous LRRK2 dephosphorylation induced by arsenite or H_2_O_2_.** *A*, lymphoblastoid cells were treated with calyculin A (10 nm, 45 min) and/or arsenite (0.5 mm), LRRK2IN-1 (2 μm), and H_2_O_2_ (2 mm), and the levels of Ser^935^ phosphorylated and total endogenous LRRK2 were assessed. *B*, quantification of Ser^935^ phosphorylation relative to total LRRK2 (one-way ANOVA; Tukey's post hoc test; *, *p* < 0.01; *n* = 2 independent experiments/condition). *C*, arsenite treatment induces the association of PP1α with LRRK2, as revealed by co-immunoprecipitation (*IP*), in HEK-293T cells transiently expressing WT FLAG LRRK2. *D*, quantification of PP1α association (one-way ANOVA; Tukey's post hoc test; *, *p* < 0.01; *n* = 3 independent experiments/condition). *Error bars*, S.E.

The differential effects of LRRK2IN-1 and arsenite stress on phosphorylation at Ser^910^/Ser^935^ of kinase-inactive D2017A suggest that the underlying mechanism does not rely on LRRK2 kinase activity. Because a recent study suggested that LRRK2 phosphorylation at Ser^910^/Ser^935^ is modulated by protein phosphatase 1 (PP1) (18), we tested whether arsenite-induced dephosphorylation was reversed by phosphatase inhibition. Calyculin A treatment of lymphoblastoid cells in the presence of arsenite, H_2_O_2_, or LRRK2IN-1 kinase inhibitor restored Ser^935^ phosphorylation of endogenous LRRK2 protein (Fig. 2, *A* and *B*). Arsenite promoted the association of LRRK2 with PP1α in a co-immunoprecipitation assay in cells transiently overexpressing FLAG LRRK2, whereas this effect was not observed for LRRK2IN-1 or H_2_O_2_ (Fig. 2, *C* and *D*). These results suggest that oxidative stress can modulate LRRK2 constitutive phosphorylation and 14-3-3 binding through a pathway that is largely independent of the kinase activity of LRRK2 but depends on PP1/PP2A phosphatase activity and that arsenite can act to stabilize the interaction of LRRK2 with PP1α.

##### Oxidative Stress Promotes LRRK2 Self-association and Assembly of Larger Complexes

**FIGURE 3.**
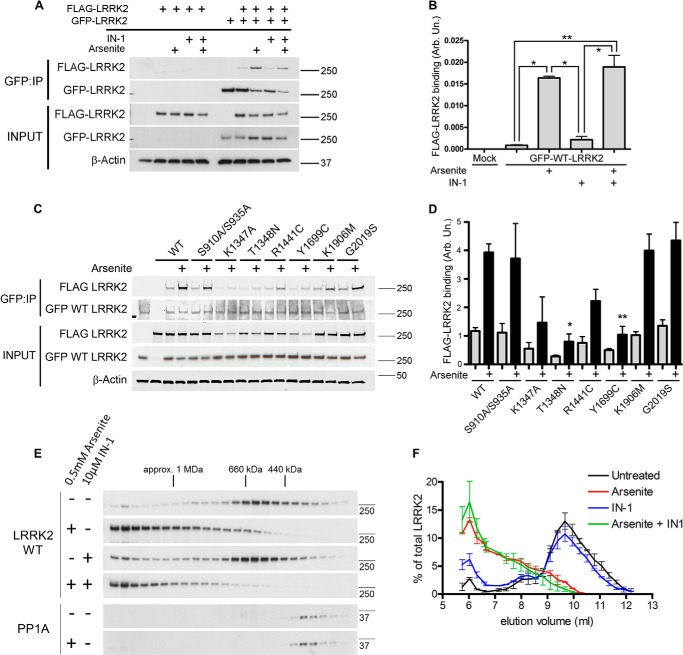
**Arsenite stress promotes LRRK2 self-association.** *A*, treatment with arsenite induces the association of FLAG LRRK2 WT with GFP LRRK2 WT when co-expressed in HEK-293T cells. *B*, quantification of FLAG LRRK2 co-immunoprecipitating with GFP LRRK2 (ratio of immunoprecipitated binding partner to the amount of GFP LRRK2 construct pulled down on the beads; one-way ANOVA; Tukey's post hoc test; *, *p* < 0.01; **, *p* < 0.001; *n* = 2 independent experiments/condition). *C*, assessment of association of mutant LRRK2 variants with WT LRRK2 under arsenite. Arsenite induces the association of LRRK2 mutants with WT LRRK2 and to a lesser extent with K1347A, T1348N, R1441C, and Y1699C mutants. *D*, quantification of LRRK2 variants co-immunoprecipitating with GFP LRRK2 (one-way ANOVA; Tukey's post hoc test; WT + arsenite *versus* T1348N + arsenite: *, *p* < 0.01; WT + arsenite *versus* Y1699C + arsenite: **, *p* < 0.05; *n* = 3 independent experiments/condition). *E*, arsenite, but not LRRK2IN-1, treatment of stably expressing HEK-293T cells promotes accumulation of high molecular mass forms of GFP LRRK2 WT on size exclusion chromatography. Arsenite stress did not alter the distribution of PP1α. Western blots of gradient fractions are shown, and approximate size markers are indicated. *F*, elution profiles of GFP LRRK2 WT after treatment of HEK-293T cells with DMSO alone (*black*), 0.5 mm arsenite (*red*), 10 μm LRRK2IN-1 (*blue*), or both arsenite and LRRK2IN-1 (*green*). *Error bars*, S.E. from duplicate independent experiments. *IP*, immunoprecipitation.

Although LRRK2 is believed to be largely monomeric in cells, it can also form dimers that may have enhanced *in vitro* enzymatic activity (7, 33–35). To investigate whether oxidative stress has an effect on the self-association of LRRK2, we performed co-immunoprecipitation using GFP-LRRK2-stably expressing HEK-293T cells co-expressing FLAG-LRRK2, with and without arsenite treatment. Arsenite treatment enhanced the self-association of LRRK2 (Fig. 3, *A* and *B*). This was not dependent on LRRK2 kinase activity because co-treatment with LRRK2IN-1 did not impede self-association.

LRRK2 dimerization is thought to be mediated by its ROC domain (8, 36, 37) in a GTP-dependent fashion (38) and is believed to be required for its enzymatic activity (33, 38, 39). To investigate the requirement of GTP binding or active kinase for the increase in self-association under arsenite, we investigated the association of WT with mutant LRRK2 variants, including the GTP binding-deficient K1347A and T1348A and the kinase-dead K1906M (Fig. 3, *C* and *D*). Arsenite enhanced the association of LRRK2 K1906M and G2019S with WT, indicating that this is not dependent on kinase activity. Strikingly, stimulation of self-association by arsenite was attenuated in the case of ROC-COR domain mutants, compared with the other variants. Based on previous reports on the involvement of the ROC-COR domain in dimerization, our data support the formation of a functional LRRK2 complex induced by arsenite treatment.

We next examined the effect of arsenite-induced stress on the apparent molecular mass of the LRRK2 complex under native conditions by size exclusion chromatography. WT LRRK2 eluted over a range of apparent molecular masses with the highest signal for immunoreactivity observed between 440 and 660 kDa, as described previously (16). Arsenite treatment shifted the elution profile of WT LRRK2 with the accumulation of very high molecular mass species (>1 megadaltons) (Fig. 3, *E* and *F*). Interestingly, kinase inhibition by LRRK2IN-1 caused only a subtle alteration in the elution of WT LRRK2 in the presence or absence of arsenite. Dephosphorylation induced by LRRK2IN-1 is insufficient for LRRK2 self-assembly (Fig. 3*A*) and the formation of large native protein complexes (Fig. 3*E*), suggesting that additional mechanisms are involved in the formation of arsenite-induced LRRK2 molecular complexes. We also investigated whether PP1α is recruited to high molecular weight species and observed that its distribution in size exclusion chromatography is not altered with arsenite treatment (Fig. 3*E*, *bottom panels*). Although this suggests that the general pool of PP1α in cells is not shifted to apparent high molecular weight fractions, it does not argue against an involvement in arsenite-induced LRRK2 dephosphorylation because PP1α is broadly distributed throughout cells, and substoichiometric amounts of this enzyme should be able to dephosphorylate LRRK2.

##### Arsenite Stress Impairs LRRK2 GTP Binding

**FIGURE 4.**
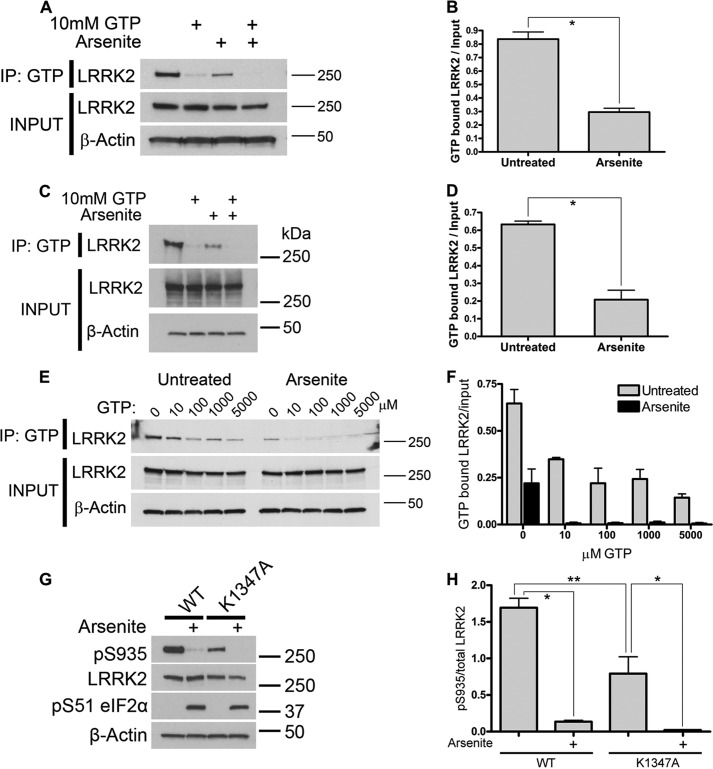
**Arsenite stress induces loss of LRRK2 GTP binding *in vitro*.** *A*, GTP binding by endogenous LRRK2 from control lymphoblasts showing input (*bottom panels*) and LRRK2 purified by binding to GTP-agarose beads. Treatment with 0.5 mm arsenite for 45 min causes a decrease in the amount of LRRK2 binding to GTP-agarose beads. *B*, quantification of the loss of GTP binding under arsenite stress (two-tailed *t* test; *p* = 0.0009; *n* = 3) by endogenous LRRK2. *C*, treatment with 0.5 mm arsenite for 45 min decreased GTP binding by GFP LRRK2 WT in stable HEK-293T cells, whereas the interaction with GTP-agarose could be disrupted by the addition of a molar excess of GTP, quantified in *D* (two-tailed *t* test; *p* = 0.0029; *n* = 3). *E*, elution of GFP LRRK2 WT from GTP-agarose by increasing concentrations of GTP. *F*, quantification of LRRK2 elution (two-way ANOVA; GTP concentration, *p* = 0.0091; arsenite, *p* < 0.0001; *n* = 2). *G*, the GTP binding-deficient LRRK2 mutant K1347A shows lower levels of Ser^935^ constitutive phosphorylation compared with LRRK2 WT when transiently expressed in cells. Arsenite treatment induced loss of K1347A Ser^935^ phosphorylation, quantified in *H* (one-way ANOVA; Tukey's post hoc test; *, *p* < 0.01; **, *p* < 0.05; *n* = 2). *IP*, immunoprecipitation. *Error bars*, S.E.

Our data suggest that arsenite can induce LRRK2 self-association (Fig. 3). Because GTP binding is thought to be important for dimerization (38), we tested whether arsenite can affect the GTP-binding properties of LRRK2. We performed *in vitro* assays using GTP-agarose to precipitate endogenous LRRK2 from lysates of lymphoblastoid cells or GFP LRRK2 from stably expressing HEK-293T cells. Arsenite treatment induced a significant decrease in the amount of LRRK2 bound to GTP-agarose at endogenous and overexpressed levels (Fig. 4, *A–D*). We performed a specific elution of LRRK2 from the GTP resin with increasing concentrations of GTP (Fig. 4, *E* and *F*). Our results are consistent with a decrease in *in vitro* GTP binding affinity for LRRK2 from arsenite-treated cells, the functional significance of which merits further investigation.

To investigate whether the effect of arsenite on Ser^935^ phosphorylation is dependent on GTP binding, we examined the K1347A GTP binding-deficient mutant (Fig. 4, *G* and *H*). When expressed in cells, K1347A LRRK2 is constitutively phosphorylated at Ser^935^ at lower levels than WT protein, suggesting that GTP binding supports this phosphorylation. However, arsenite stress was capable of diminishing Ser^935^ phosphorylation of K1347A LRRK2, suggesting that GTP binding is not required for arsenite-induced dephosphorylation.

##### Arsenite Attenuates in Vitro Kinase Activity of LRRK2

**FIGURE 5.**
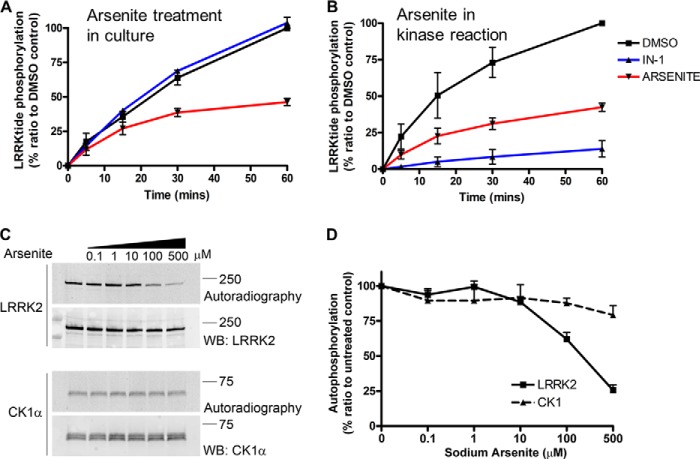
**Arsenite attenuates the kinase activity of LRRK2 *in vitro*.** *In vitro* kinase assays using LRRK2 WT protein and LRRKtide peptide as a LRRK2 substrate were performed, measuring the incorporation of [γ-^33^P]ATP in a time course. In *A*, cells overexpressing FLAG LRRK2 WT were untreated or treated with arsenite (500 μm) or LRRK2IN-1 (10 μm), and FLAG LRRK2 was subsequently purified and used in the LRRKtide phosphorylation assay. Treatment with arsenite prior to protein purification reduced LRRKtide phosphorylation by LRRK2 *in vitro*, whereas LRRK2IN-1 did not inhibit kinase activity because it is removed during the purification process (*error bars*, S.E. from *n* = 2 independent experiments). In *B*, FLAG LRRK2 WT protein was purified from transiently expressing untreated HEK-293T cells and used in kinase assays, where the addition of sodium arsenite (500 μm) in the reaction significantly reduced LRRKtide phosphorylation. LRRK2IN-1 inhibited LRRK2 *in vitro* kinase activity (*n* = 3 independent experiments/condition). *C*, autophosphorylation of recombinant GST-LRRK2(970–2529) and GST-CK1α in the presence of increasing concentrations of sodium arsenite. *D*, quantification of the effect of increasing concentrations of arsenite on the autophosphorylation of LRRK2 or CK1α (*error bars*, S.E. from *n* = 3 independent experiments).

LRRK2 possesses an active kinase domain, the activity of which is reportedly influenced by some genetic mutations, dimerization, and GTP binding. Our data indicate that arsenite can alter LRRK2 self-association and GTP binding while also modulating constitutive phosphorylation at Ser^910^/Ser^935^ and affecting the autophosphorylation site Thr^1410^. We therefore tested the hypothesis that arsenite can affect the kinase activity of LRRK2, using an *in vitro* assay system. HEK-293T cells expressing FLAG LRRK2 were treated with LRRK2IN-1 or arsenite, and LRRK2 was purified and assayed for kinase activity, measuring the incorporation of ^33^P with the model substrate LRRKtide (29). We found that whereas pretreatment with LRRK2IN-1 did not have a measurable effect on LRRK2 kinase activity, presumably because the inhibitor is washed off at the protein purification steps, pretreatment with arsenite (500 μm) attenuated the kinase activity of LRRK2 (Fig. 5*A*). We subsequently tested whether arsenite can directly inhibit LRRK2 when added to the kinase reaction. FLAG LRRK2 WT was purified from untreated cells and used in kinase assays with LRRK2IN-1 or arsenite (Fig. 5*B*). Whereas LRRK2IN-1 (10 μm) abolished LRRK2 kinase activity, arsenite (500 μm) decreased the incorporation of ^33^P on LRRKtide by ∼50% compared with untreated controls at 60 min of incubation. To address the specificity of the effect on LRRK2, an *in vitro* autophosphorylation kinase assay was performed with recombinant LRRK2 and an unrelated kinase casein kinase 1α (CK1α) in the presence of increasing concentrations of arsenite (Fig. 5, *C* and *D*). Arsenite (500 μm) inhibited LRRK2 autophosphorylation, whereas CK1α autophosphorylation was largely unaffected (Fig. 5*D*). These results suggest that arsenite may have a direct effect on LRRK2 activity.

##### Arsenite Stress Induces LRRK2 Ubiquitylation and Translocation to Centrosomes

**FIGURE 6.**
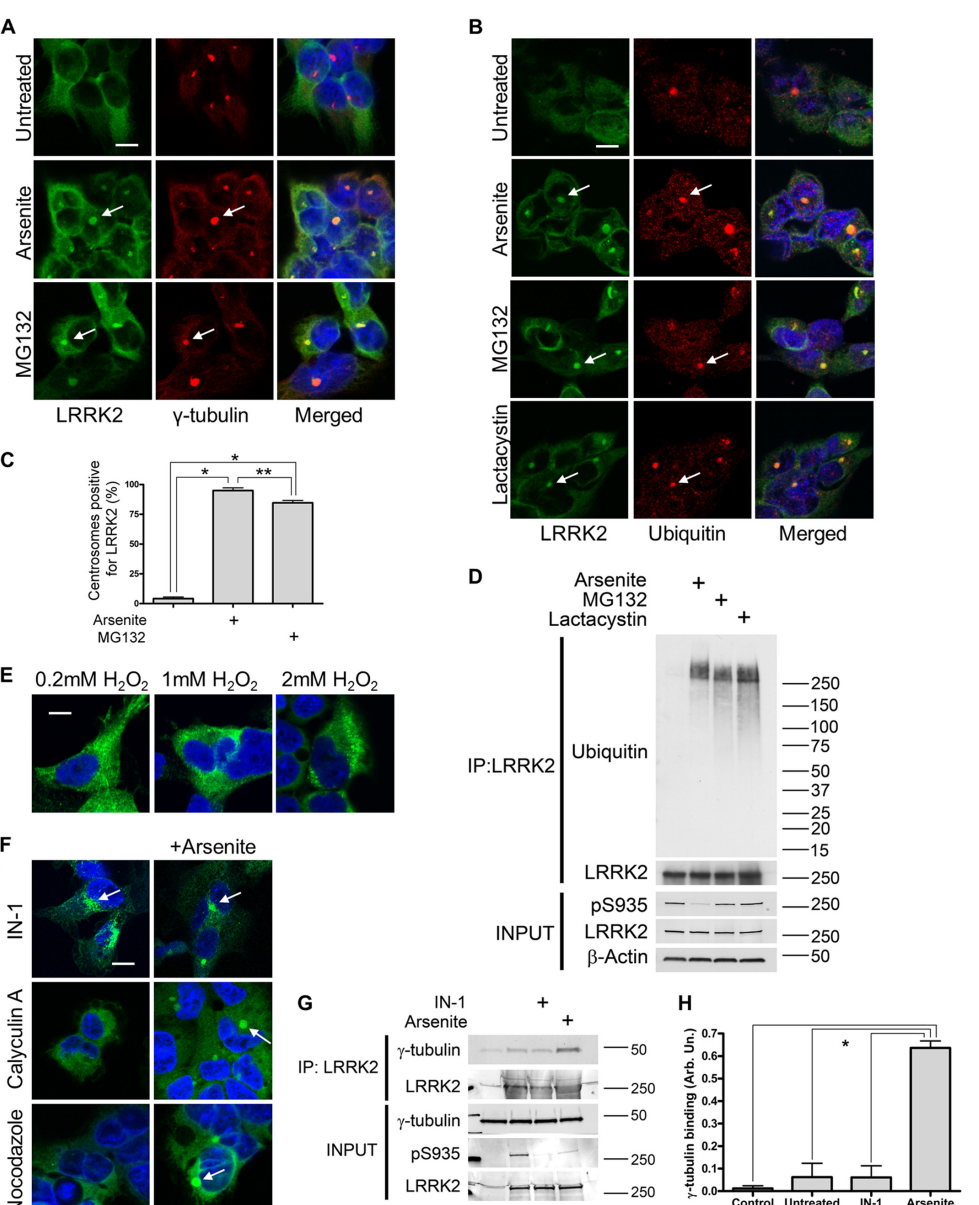
**Arsenite stress promotes ubiquitylation of GFP-LRRK2 and translocation to centrosome proteolytic center.** Acute arsenite treatment or proteasomal inhibition induces accumulation of GFP-LRRK2 in perinuclear bodies co-localizing with γ-tubulin staining of centrosomes (*A*) and ubiquitin immunostaining (*B*). *C*, quantification of MG132-induced translocation of LRRK2 to centrosomes (300 cells were counted per treatment in two independent experiments; one-way ANOVA; Tukey's post hoc test; *, *p* < 0.001; **, *p* < 0.01). *Scale bar*, 10 μm. *D*, immunoprecipitated (*IP*) GFP LRRK2 WT from untreated or arsenite-, MG132-, or lactacystin-treated cells was probed for ubiquitin. Arsenite or proteasomal inhibitor treatment promotes ubiquitylation of LRRK2. Arsenite, but not lactacystin or MG132, induced loss of Ser^935^ dephosphorylation. *E*, treatment with increasing concentrations of H_2_O_2_ does not induce translocation of LRRK2 to centrosomes. *F*, translocation of LRRK2 to ubiquitin-positive centrosomes under arsenite treatment is independent of LRRK2 kinase activity, as shown by co-treatment with LRRK2IN-1 (*i*); does not require Ser^910^/Ser^935^ dephosphorylation under arsenite, as suggested by co-treatment with calyculin A (*ii*); and is not inhibited by depolymerization of the microtubule network by nocodazole (*ii*). *G*, arsenite stress induces association of LRRK2 with γ-tubulin. *H*, quantification of γ-tubulin association (one-way ANOVA; Tukey's post hoc test; *, *p* < 0.05; *n* = 2 independent experiments/condition). *Scale bar*, 10 μm.

LRRK2 is largely cytoplasmic but can also be associated with membranous structures (40, 41). Loss of Ser^910^/Ser^935^ phosphorylation, triggered by kinase inhibition, alters the distribution of LRRK2 and promotes the formation of skein-like structures (10, 13, 15), a phenomenon that is not observed with kinase-dead LRRK2 (16). To examine the effects of oxidative stress on the subcellular localization of LRRK2, we treated GFP-LRRK2 HEK-293T cells with arsenite followed by confocal microscopy. In the absence of treatment, WT LRRK2 showed diffuse cytosolic staining. Upon arsenite stress, WT LRRK2 localized in discrete perinuclear pools (Fig. 6*A*). Because there was only a single area of LRRK2 staining in each cell, we addressed whether these structures were related to centrosomes. Co-staining with γ-tubulin revealed strong co-localization of LRRK2 with large perinuclear centrosomes under arsenite treatment.

The centrosome represents the cellular microtubule-organizing center, but it is also believed to be a center for proteolysis and protein folding, where factors involved in the ubiquitin proteasome pathway (UPS) are sequestered and associate with γ-tubulin (42, 43). LRRK2 can be degraded via the ubiquitin proteasome pathway (44) or by chaperone-mediated autophagy (45). To investigate the involvement of the UPS in LRRK2 sequestration to the centrosome, we inhibited proteasome activity by MG132 and examined the localization of LRRK2. MG132 induced translocation of LRRK2 to centrosomes to a degree similar to that observed with arsenite (Fig. 6, *A* and *C*). Double staining with an anti-ubiquitin antibody revealed that LRRK2 is sequestered to ubiquitin-positive perinuclear centrosomes after acute arsenite treatment or 6-h treatment with MG132 or lactacystin (Fig. 6*B*). We further investigated whether LRRK2 is itself polyubiquitinated, by immunoprecipitation followed by Western blotting, which revealed that acute arsenite treatment induces ubiquitylation of GFP LRRK2 WT in cells (Fig. 6*D*). Similar effects were seen with proteasomal inhibition by either MG132 or lactacystin. Previous studies have reported that arsenite can induce accumulation of ubiquitylated proteins (46, 47). MG132 or lactacystin did not have a significant effect on Ser^935^ phosphorylation of LRRK2 (Fig. 6*D*), suggesting that arsenite-induced dephosphorylation is not required for translocation of LRRK2 to centrosomes.

To test whether oxidative stress is sufficient to drive LRRK2 intracellular translocation, we treated WT LRRK2-expressing cells with increasing concentrations of H_2_O_2_. This did not alter the localization of LRRK2, indicating that increased cytosolic reactive oxygen species is not sufficient for centrosomal association (Fig. 6*E*). This also supports the proposition that loss of Ser^910^/Ser^935^ phosphorylation is not sufficient to drive translocation to centrosomes. We next examined whether kinase activity is required for centrosomal association after arsenite treatment. Kinase inhibition by LRRK2IN-1 produced skeinlike structures, as reported previously (18); however, this did not rescue translocation to centrosomes under arsenite co-treatment (Fig. 6*F*).

We further addressed whether loss of phosphorylation is required for WT LRRK2 translocation to centrosomes by treating with calyculin A prior to arsenite stress. Arsenite stress induced LRRK2 translocation to centrosomes even under calyculin A co-treatment, suggesting that loss of Ser^910^/Ser^935^ phosphorylation is neither sufficient nor required for translocation of LRRK2 into centrosomes under arsenite stress (Fig. 6*F*).

Microtubules that originate at the microtubule-organizing center mediate retrograde transport of vesicles and organelles. LRRK2 is believed to interact with microtubules through its ROC domain (48, 49). Depolymerization of the microtubule network by nocodazole prior to arsenite treatment did not block the translocation of LRRK2 to centrosomes by arsenite (Fig. 6*F*). Depolymerization of microtubules also did not alter arsenite-induced WT LRRK2 Ser^935^ dephosphorylation (data not shown).

We further investigated the recruitment of LRRK2 to centrosomes by examining a possible association with γ-tubulin. In co-immunoprecipitation experiments, arsenite treatment enhanced the association of GFP LRRK2 WT with endogenous γ-tubulin (Fig. 6, *G* and *H*). These data suggest that recruitment of LRRK2 to the centrosome is not dependent on microtubule-associated transport mechanisms but may be influenced by association with γ-tubulin.

##### Effect of LRRK2 Mutations on Centrosomal Association

**FIGURE 7.**
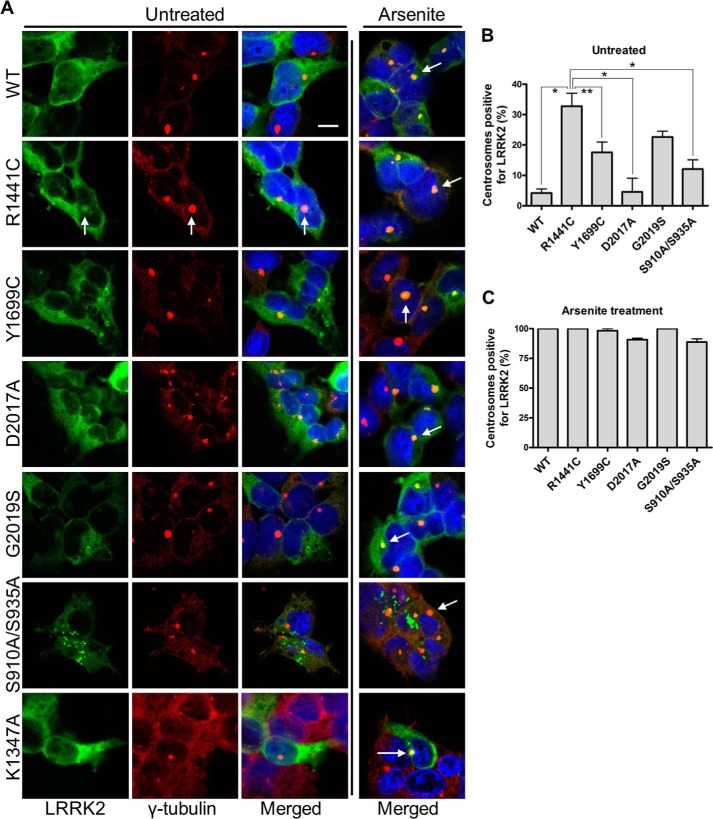
**LRRK2 mutations and association with centrosomes.** *A*, inducible HEK-293T cells expressing WT or genetic mutants of LRRK2 and HEK-293T cells transiently expressing the GTP binding-deficient mutant K1347A LRRK2 (*bottom panels*) were treated with sodium arsenite followed by immunostaining for γ-tubulin. The introduction of arsenite induced translocation of LRRK2 to centrosomes in all genetic variants examined. R1441C LRRK2 showed partial co-localization with centrosomes in the absence of arsenite stimulation. Centrosomes were counted for positivity for LRRK2, and data are presented as bar graphs for untreated (*B*) and arsenite-treated cells (*C*) (100 cells were counted per genetic variant and treatment; one-way ANOVA; Tukey's post hoc; *, *p* < 0.01; **, *p* < 0.05). *Scale bar*, 10 μm. *Error bars*, S.E.

We also investigated the effect of LRRK2 mutations on the sequestration of LRRK2 to centrosomes in cells stably expressing mutant forms of LRRK2 (Fig. 7). In contrast to WT LRRK2, in unstressed cells, R1441C LRRK2 displayed some perinuclear staining co-localizing with γ-tubulin, with ∼30% of centrosomes positive for R1441C LRRK2 (Fig. 7, *A* and *B*). Perinuclear localization of the Y1699C and G2019S variants was evident in ∼20% of centrosomes in stable expression cell lines, whereas D2017A exhibited only minimal centrosomal localization. The S910A/S935A mutant that cannot be phosphorylated did not co-localize with centrosomes in the absence of treatment (Fig. 7*A*). Arsenite treatment induced accumulation of LRRK2 WT and variants into centrosomes stained for γ-tubulin with >95% of centrosomes positive for LRRK2 in WT as well as the R1441C, Y1699C, D2017A, and G2019S (Fig. 7, *A* and *C*). However, the S910A/S935A mutant produced an intermediate phenotype following arsenite treatment, localizing into centrosomes as well as uncharacterized cytoplasmic foci. This suggests that whereas dephosphorylation may dynamically mobilize LRRK2, arsenite-induced stress invokes additional cell signaling events driving LRRK2 translocation to centrosomes.

To examine the requirement of GTP binding for the shift in localization following arsenite treatment, we transiently expressed LRRK2 K1347A in HEK-293T cells (Fig. 7*A*, *bottom panels*). LRRK2 K1347A accumulated in centrosomes under arsenite treatment, suggesting that GTP binding is not required for arsenite-induced association of LRRK2 with centrosomes. The kinase-dead D2017A mutant was also recruited to centrosomes (Fig. 7*A*); thus, centrosomal association is independent of the kinase activity of LRRK2. Collectively, these data suggest that the translocation of LRRK2 to centrosomes under arsenite is probably related to degradation of the protein by the UPS, and the pathway is independent of the activity of LRRK2 itself.

## DISCUSSION

Oxidative stress is implicated in Parkinson disease with pathology involving several genes associated with mitochondrial function and integrity (reviewed in Ref. 19). Although many interacting partners of LRRK2 and potential functions have been described, including roles in autophagy, regulation of the cytoskeleton, neurite outgrowth, and regulation of microRNA, the physiological regulation of LRRK2 in cell signaling is unclear. Here we report that oxidative stress induced by either arsenite or H_2_O_2_ treatment caused dephosphorylation of LRRK2 Ser^910^ and Ser^935^ with concomitant loss of 14-3-3 binding, hence mobilizing LRRK2 within the cell. Furthermore, arsenite stress induced an increase in self-association with a reduction in GTP binding *in vitro*, formation of macromolecular complexes, and translocation to centrosomes.

LRRK2 is a serine/threonine kinase that is phosphorylated by other largely unknown kinases. A number of autophosphorylation sites have also been identified in the ROC domain and the kinase domain (50, 51). Phosphorylation at specific sites can affect the enzymatic function and modulate the pathogenic cellular effects of PD mutations (52–54). Amino acids Ser^910^ and Ser^935^ are not autophosphorylation sites (14). Nonetheless, it is known that LRRK2 inhibitor LRRK2IN-1 causes marked dephosphorylation of these sites (13), although the precise mechanism(s) involved has not yet been defined. Our results indicate that arsenite and H_2_O_2_-induced stress promote dephosphorylation of these sites independently of LRRK2 kinase activity (Fig. 1), possibly mediated by phosphatase PP1 (18). Arsenite treatment enhances PP1α binding to LRRK2, suggesting that it stabilizes this otherwise transient interaction. Furthermore, arsenite can reportedly inhibit IκB activation (55), whereas the IκB kinase family can mediate LRRK2 Ser^910^/Ser^935^ phosphorylation (17). It is possible that direct inhibition of IκB kinase by arsenite could also contribute to LRRK2 dephosphorylation. Calyculin A (PP1/PP2A inhibitor) rescues dephosphorylation induced by arsenite and H_2_O_2_, but only arsenite induces increased PP1α association under these conditions. It would be interesting to investigate whether other phosphatases are involved in dephosphorylation events induced by H_2_O_2_. The fact that kinase-dead LRRK2 responds to arsenite stress with loss of Ser^910^/Ser^935^ phosphorylation but is not affected by pharmacological kinase inhibition suggests that dephosphorylation is mediated by distinct signaling events and perhaps reflects arsenite-induced conformational changes in LRRK2 that render the phosphorylation sites accessible to phosphatases.

Phosphorylation at Ser^910^/Ser^935^ mediates 14-3-3 binding to LRRK2 (13). 14-3-3 can control the localization of target proteins by steric hindrance of protein interactions (reviewed in Ref. 56). Recently, 14-3-3 has been shown to inhibit the function of GTP-binding Rnd proteins by sequestering them in the cytosol (57). It is possible that 14-3-3 binding inhibits LRRK2 interactions with specific proteins and prevents its activation by maintaining a cytosolic location. Arsenite and H_2_O_2_-induced Ser^910^/Ser^935^ dephosphorylation promotes 14-3-3 dissociation that allows LRRK2 to be relocated (15); however, additional factors are likely to participate in determining the final subcellular destination.

We observe an increase in LRRK2 self-association with arsenite treatment, indicating dimer formation possibly mediated by the ROC domain (37). Our data show that although arsenite induces the association of WT LRRK2 with WT and the genetic variants G2019S or kinase-dead K1906M LRRK2, this effect is more modest in the case of the variants that affect GTP binding and/or GTP hydrolysis (Fig. 3*C*). This implies that arsenite-induced self-interaction may be dependent on GTP binding capacity. This is in accordance with a recent study suggesting that LRRK2 mutations that impair guanine nucleotide binding (K1347A and T1348N) attenuated the formation of dimers (38). This may have important implications in structural studies of LRRK2; if arsenite stabilizes a LRRK2 self-complex *in vitro*, it may prove an invaluable tool in modulating LRRK2 complex formation. Arsenite treatment induced accumulation of LRRK2 in >1-MDa species. Previous reports indicate that monomeric forms of WT LRRK2 sediment in unexpected high molecular mass fractions (∼1.3 MDa) (41, 58), whereas a kinase-dead LRRK2 variant shows a similar high apparent mass in FPLC (7). Although the nature of the apparent size shift with arsenite remains to be investigated, taken together with the increase in self-association, it suggests sequestration into protein complexes supporting activation of LRRK2 signaling pathway(s).

Our results indicate that arsenite treatment impairs the GTP binding properties of LRRK2 *in vitro*. It is possible that the loss of *in vitro* GTP binding following arsenite treatment is the result of the formation of a tight LRRK2 dimer or stable protein complex rendering the ROC domain inaccessible *in vitro* to GTP-agarose. Another possibility is that LRRK2 is GTPase-active, consistent with arsenite inducing formation of a dimer (39). GTP may still be hydrolyzable on the GTP-agarose beads used in our *in vitro* assays because conjugation to agarose is not through the γ-phosphate. In this case, it is possible that an increased rate of GTP hydrolysis by LRRK2 may be detected as lower stable binding to GTP in this assay.

It has been postulated that the arsenic ion arsenate (AsO_4_^3−^) can substitute phosphate in certain biological reactions (59). Although in our experiments, we use sodium meta-arsenite (NaAsO_2_), arsenate contaminants may potentially compete with phosphate in our *in vitro* kinase reactions. The fact that (*a*) autophosphorylation of the unrelated kinase CK1α is not affected *in vitro* and (*b*) arsenite treatment does not globally inhibit kinases (eIF2α is hyperphosphorylated following arsenite treatment; Fig. 1) suggest that this is a specific effect. However, the kinase inhibition requires relatively high concentrations of arsenite; therefore, the biological significance of this merits further investigation. Nevertheless, arsenite inhibits the activation of IκB kinase by binding to the cysteine 179 residue in the activation loop of its catalytic subunits (55, 60). It would therefore be interesting to see whether arsenite affects LRRK2 kinase activity by direct binding to its kinase catalytic core.

We show that arsenite treatment also causes LRRK2 to relocalize to the centrosome, co-localizing with γ-tubulin. Although pharmacological kinase inhibition by LRRK2IN-1 also induces dephosphorylation, this can result in translocation of LRRK2 to microtubule-like skein structures. Furthermore, S910A/S935A LRRK2, which is incapable of phosphorylation, exhibits a further distinct phenotype (Fig. 7). Thus, the loss of Ser^910^/Ser^935^ phosphorylation is not the only factor mediating LRRK2 translocation. γ-Tubulin has been linked to sequestration of UPS factors (42, 43). In our experiments, proteasomal inhibition also induced centrosomal accumulation of LRRK2, whereas arsenite was shown to induce LRRK2 ubiquitylation. These data suggest that accumulation to centrosomes is mediated by protein degradation machinery (Fig. 7*C*). Interestingly, the ROC domain mutant R1441C shows some co-localization with centrosomes in untreated conditions. The fact that arsenite treatment produces a phenotype in all variants that is similar to the effect of this ROC domain mutation may suggest changes in LRRK2 conformation and autoinhibition. LRRK2 has been reported to localize in perinuclear bodies described as aggresomes (61) that can associate with γ-tubulin. However, we did not observe co-localization of LRRK2 with vimentin, an aggresome marker (62) (data not shown).

Our data suggest that signaling of the cellular response to arsenite stress directly regulates LRRK2. Arsenite disrupts ATP production by inhibiting pyruvate dehydrogenase and induces generation of nitric oxide and reactive oxygen species in cells partly by mediating the increase in intracellular H_2_O_2_ levels (63–65). It also induces translation arrest while mediating formation of cytoplasmic stress granules that contain mRNA and components of the translation machinery (66). The various biochemical effects of arsenite raise the possibility that the specific pathway leading to LRRK2 may be the convergence of multifactorial events. This may explain why pure oxidative stressors have not been reported to have similar effects on LRRK2.

**FIGURE 8.**
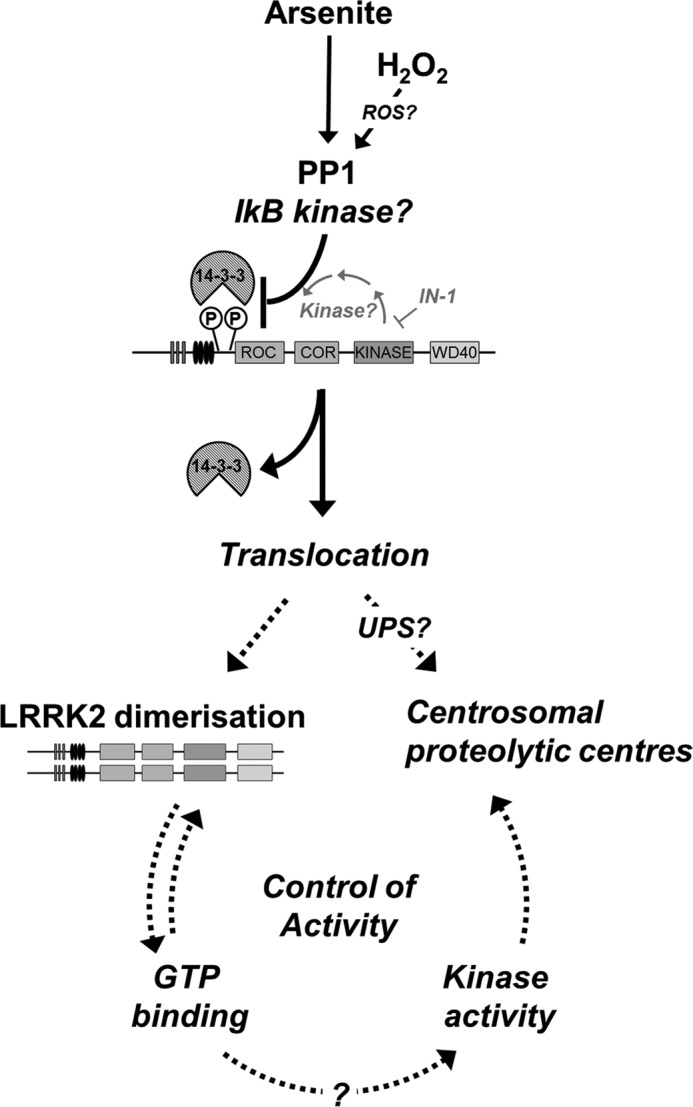
**A model of the modulation of LRRK2 by arsenite and H_2_O_2_.** LRRK2 is constitutively phosphorylated at Ser^910^/Ser^935^, and its kinase activity can modulate this through a signal loop pathway involving other kinases, whereas phosphorylation is inhibited by kinase inhibition. Arsenite or H_2_O_2_ induces the loss of Ser^910^/Ser^935^ phosphorylation through PP1 in signaling events independent of the kinase activity of LRRK2. This induces dissociation from 14-3-3 and mobilization promoting formation of LRRK2 dimers that may be assembled into larger protein complexes. Modulation of its dimerization is linked to altered GTP binding and influences substrate binding and kinase activity. Oxidative stress signaling ultimately controls the balance between activation of LRRK2 and the removal of LRRK2 protein by UPS.

We propose a model describing arsenite-induced oxidative stress regulation of LRRK2 (Fig. 8). Monomeric LRRK2 is largely cytosolic, and phosphorylation at Ser^910^/Ser^935^ is controlled by upstream kinases as well as a feedback loop pathway dependent on its kinase activity. Arsenite and H_2_O_2_ induce dephosphorylation of LRRK2 by stimulating phosphatases, such as PP1, and/or by modulating upstream kinase activity, such as IκB, causing dissociation from 14-3-3. This mobilizes and translocates LRRK2 to membranes, where it assembles into LRRK2 complexes (Fig. 8). Modulation of its self-association and GTP binding influences its kinase activity and association with substrates. Arsenite-induced signaling may ultimately control the critical balance between activation of LRRK2 and removal by UPS.

In summary, our data show that two oxidative stressors, H_2_O_2_ and arsenite, affect LRRK2 in several different ways that would be consistent with protein activation, namely dephosphorylation, formation of oligomeric species, and altered binding of GTP. However, arsenite, which can inhibit LRRK2 kinase directly, causes accumulation of LRRK2 at the centrosome, probably for protein degradation. We speculate that the difference between H_2_O_2_ and arsenite suggests that cells will remove an inactive LRRK2 molecule, which may be relevant for termination of cellular signaling by LRRK2 protein complexes. We therefore propose that the balance between activation of LRRK2 by oxidative stress and its degradation by the UPS is a critical mediator of protein function.

## Footnotes

4 The abbreviations used are:

PD: Parkinson disease
LRRK2: leucine-rich repeat kinase 2
TRITC: tetramethylrhodamine isothiocyanate
ANOVA: analysis of variance
PP1 and PP2: protein phosphatase 1 and 2, respectively
CK1α: casein kinase 1α
UPS: ubiquitin proteasome pathway.
